# A brief nap during an acute stressor improves negative affect

**DOI:** 10.1111/jsr.13701

**Published:** 2022-07-18

**Authors:** Nathan Wofford, Natalie Ceballos, Gary Elkins, Carmen E. Westerberg

**Affiliations:** ^1^ Department of Psychology Texas State University San Marcos Texas USA; ^2^ Department of Psychology and Neuroscience Baylor University Waco Texas USA

**Keywords:** cognition, negative affect, sleep, stress, working memory

## Abstract

Overnight sleep can reduce perceived stress, and improve associated cognitive disruptions and negative affect after an acute stressor. Whether a brief nap can also bestow these benefits in a non‐sleep‐restricted population is currently unknown. In this study that used a between‐subjects design, stress was triggered by administering a modified Trier Social Stress Test to two groups of participants (nap [*n* = 29], wake [*n* = 41]). All participants were instructed they would give a speech during the study but the topic would be withheld until later, and then completed a math task. After a 40‐min break in which participants watched a neutral video or took a nap monitored with electroencephalography, stress was reinforced by presenting the speech topics and giving participants a 10‐min preparation period. Next, instead of giving a speech, the study ended and participants were debriefed. Negative affect, perceived stress and working memory were measured at multiple time points before and after the break. Both groups showed lower perceived stress and improved working memory after the break than before, but a nap did not confer additional benefits for perceived stress or working memory beyond taking a break. However, the nap group exhibited lower negative affect after the break than the wake group, and only the nap group showed a reduction in negative affect compared with initial negative affect levels. These results indicate a nap can improve negative emotions accompanying a stressor to a greater extent than taking a break, and suggest that brief naps may be a useful way to improve mood while experiencing an acute stressor.

## 1 INTRODUCTION

The average healthy adult gets 7–9 hr of sleep every night (Hirshkowitz et al., 2015). While spending a third of one's life unconscious and inactive is costly, failing to get sufficient high‐quality sleep has dire effects on physiological and psychological health (Goldstein & Walker, 2014; Irwin, 2015). Physiologically, poor sleep quality is associated with overweight and obese status, physical health problems, and impaired immune function (Fatima et al., 2016; Pilcher et al., 1997; Stein et al., 2008). Furthermore, sleep disturbance can reduce cytokine production, and in turn lead to increased susceptibility to viruses (Irwin, 2015). Regarding mental health, poor sleep quality is associated with increased symptoms of anxiety and depression, increased emotional reactivity and negative affect, and reductions in life satisfaction, whereas adequate sleep promotes adaptive emotional responding (Goldstein & Walker, 2014). Reductions in sleep quality also have negative consequences for cognition, including impairments in working memory (Alhola & Polo‐Kantola, 2007).

The contribution of sleep to working memory deserves particular attention due to the reliance of many cognitive abilities on optimal working memory function, including decision‐making, reasoning, attention, learning and multi‐tasking, among others, that are essential for completing daily living activities. Sleep deprivation has been shown to dramatically reduce working memory performance (Chee & Choo, 2004; Van Dongen et al., 2003), and acoustic augmentation of slow waves during sleep has been shown to improve working memory in individuals who responded to the acoustic stimulation (Diep et al., 2020), suggesting a causal role of sleep for working memory function.

Sleep also plays a vital role in managing stress responses, which typically entail physiological, psychological and cognitive components (Schoofs et al., 2008). Poor sleep has been associated with exaggerated responses to stressors, including heightened cortisol reactivity, increases in both subjective stress and negative affect (Minkel et al., 2012; Mrug et al., 2016), and impaired working memory (Alhola & Polo‐Kantola, 2007). On the other hand, good sleep quality has been associated with decreased perceived stress, changes in cortisol reactivity, and improvements in working memory (Bassett et al., 2015; Kuriyama et al., 2008; Wu et al., 2015).

Sleep is typically divided into four stages: stage 1; stage 2; slow‐wave sleep (SWS); and rapid eye movement (REM) sleep. Importantly, psychological, physiological and cognitive aspects of the stress response have been linked with multiple aspects of sleep. SWS and associated slow‐wave activity (SWA; 0.5–1.5 Hz oscillations predominant during SWS) are thought to be important for downscaling synaptic strengths, allowing over‐loaded synapses to reset, which facilitates experience‐dependent plasticity during waking (Tononi & Cirelli, 2006). Several recent studies suggest that SWS may also enable optimal cognitive performance (Van Der Werf et al., 2011). Therefore, these restorative aspects of SWS may be especially useful for overcoming the psychological and cognitive deficits associated with acute stress responses.

Yet, REM may also play a role in reducing stress responses. While hypothalamic–pituitary–adrenal (HPA) axis activity is suppressed during SWS, this inhibition is released during REM, resulting in relatively higher blood cortisol concentrations during REM than other sleep stages (Born & Fehm, 1998). In addition, both adrenaline and noradrenaline levels are reduced during overnight REM (Dodt et al., 1997). This reduction may be useful for recalibrating responsiveness to future stressful events and reducing the emotional tone associated with prior stressful events (Goldstein & Walker, 2014). Supporting this idea, reductions in REM have been associated with increased subjective state anxiety, a common index of perceived stress, increased levels of noradrenaline, and decreased working memory (Lau et al., 2015; Mallick & Singh, 2011; Motomura et al., 2017). Thus, it is currently unclear if SWS is sufficient to reduce stress responses, or if REM or a combination of both SWS and REM are necessary for reductions.

Given the importance of sleep for both physical and mental health, daytime napping may provide a means for individuals who do not get sufficient overnight sleep to ward off potential physical and mental health problems. Napping has been shown to have a host of physiological and psychological benefits. For example, a 30‐min nap after a sleep‐deprived night can return cortisol and leukocyte levels, which act as biomarkers of inflammatory processes and immune function, back to baseline levels (Faraut et al., 2011). Consistent midday naps can also help regulate emotions by increasing tolerance for frustration and reducing negative affect (Goldschmied et al., 2015; Jones et al., 2019). Additionally, naps have been shown to improve logical reasoning, reaction time, long‐term memory and working memory (MacDonald et al., 2018; Milner & Cote, 2009).

Although it is clear from the existing literature that, like overnight sleep, napping can provide physical, psychological and cognitive benefits, especially for those who are sleep deprived, the potential for a short nap to mediate the negative effects concomitant with acute stressors is unknown. Brief naps (< 60 min) typically do not allow individuals to achieve REM sleep, given that it takes 90–110 min to cycle through the four sleep stages and REM is the final stage achieved during each cycle. Thus, if REM in particular is responsible for ameliorating stress and improving affect and cognition, a brief nap may not be useful. However, it is also possible that the role of different sleep stages in ameliorating stress responses may differ between overnight sleep and sleep during a nap due to circadian or other factors, as has been observed in other domains. For example, emotional memory consolidation has been associated with REM during overnight sleep but with SWS during napping (Alger et al., 2018). An additional complicating factor is that stress may diminish SWA during a nap. Ackermann et al. (2019) found that after a psychosocial stressor, sleep latency increased and SWA was reduced during the first 30 min of a 90‐min nap. Furthermore, napping after acute stress did not influence long‐term memory or vigilance relative to a wake control group. However, perceived stress, affect and other aspects of cognition were not measured.

Given the uncertainties surrounding how sleep and napping in particular may influence perceived stress, affect and cognition during an acute stressor, the purpose of this study was to determine how a brief 40‐min nap during an acute stressor may influence the psychological and cognitive components of the stress response in a non‐sleep‐restricted population. After exposure to an acute stressor, participants took a break that included either a 40‐min nap or a comparable time spent awake, and perceived stress, negative affect and working memory were measured both before and after the break for both groups to determine how a brief nap may influence these measures. Following the break, participants were reminded of the stressor, they were given 10 min to prepare their speeches, and negative affect and perceived stress were measured for a final time to investigate whether the nap conferred any lasting benefits for negative affect and perceived stress. Potential sex differences in perceived stress and negative affect were also examined, as women can have more negative emotional responses to stressors than men (Kelly et al., 2008).

## 1 METHODS

### 1.1 Participants

**FIGURE 1:**
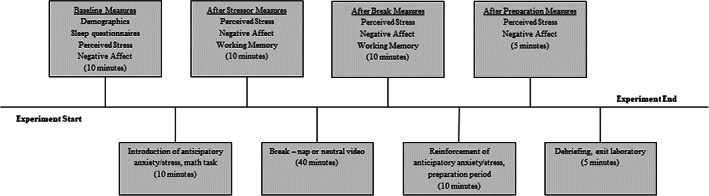
Timeline of events during the laboratory testing session. Times listed denote approximate durations for each task

This study was approved by the Texas State University Institutional Review Board. Participants were recruited to take part in a study measuring the relationship between sleep, stress and memory from undergraduate psychology courses at Texas State University, and were compensated with extra credit or $20. All participants provided written informed consent. Participants were randomly assigned to either the nap group or wake group prior to their arrival, and all testing sessions began between 12:00 hours and 16:00 hours (Figure 1). The final sample comprised 29 nap participants and 41 wake participants (mean age = 20.31 years, 35 male), which is comparable to Jones et al. (2019), who measured how naps influence mood. Twenty‐seven additional nap participants were excluded for failure to achieve 5 min of stable sleep. Nine additional wake participants withdrew after beginning the study.

### 1.2 Materials

Sleep quantity and quality for the previous night and previous month were assessed using the Karolinska Sleep Diary (KSD) and the Pittsburgh Sleep Quality Index (PSQI), respectively (Åkerstedt et al., 1994; Buysee et al., 1989). The KSD includes six questions regarding sleep quantity and seven questions about perceived sleep quality, in which responses are recorded on a 1–5 scale (Table 1). The PSQI includes 19 questions related to sleep quantity and quality, and a global score is calculated, ranging from 0 to 21, with scores > 5 indicating increasingly poor sleep quality.

**TABLE 1:** KSD questions

Sleep quantity questions	Sleep quality questions
At what time did you go to bed and turn the lights off last night? At what time did you arise this morning? How long did you sleep? How long did it take you to fall asleep? How many awakenings did you have last night? How many total minutes were you awake after falling asleep last night?	7 How did you sleep? 8 Did you feel refreshed after you arose this morning? 9 Did you sleep soundly? 10 Did you sleep throughout the time allotted for sleep? 11 How easy was it for you to wake up? 12 How easy was it for you to fall asleep? 13 How much did you dream last night?

State anxiety, an index of perceived stress (Lu et al., 2016), was measured with the Spielberger State Anxiety Inventory (SSAI; Spielberger et al., 1983). Participants rated 20 statements based on how much they currently identified with it on a 1–4 scale (*1 = not at all; 4 = very much so*). Half of the questions corresponded to stressful/anxious moods (e.g. I am tense, I am nervous), and half corresponded to non‐stressful/non‐anxious moods (e.g. I feel calm, I feel pleasant). Ratings were then combined such that higher scores indicated higher levels of stress.

Negative affect was measured using the Positive and Negative Affect Schedule (PANAS; Watson et al., 1988). Participants viewed 20 words, and were asked to rate how much each word describes how they were currently feeling on a 1–5 scale (*1 = very slightly or not at all; 5 = extremely*). Half of the words corresponded to positive affect (e.g. interested, excited, enthusiastic) and half corresponded to negative affect (e.g. distressed, irritable, afraid). Negative affect scores were calculated by summing the ratings for the negative words.

Working memory was measured using the Digit Span‐Backwards subtest from the Wechsler Adult Intelligence Scale (WAIS; Wechsler, 2008). Participants were read aloud number sequences and were asked to immediately report the sequence back in reverse order. The number of digits in each sequence increased from 2 to 8 digits. There were 16 sequences, and 1 point was given for each correct sequence report.

### 1.3 Modified Trier Social Stress Test (TSST)

To induce acute stress, participants completed a modified version of the TSST that was divided into two phases. During Phase 1, participants were told they would be taking on the role of a job applicant and would have to give a speech to a selection committee, but they were not given their specific speech topic. This was done to maintain stress throughout the study and to ensure that participants were not planning their speech throughout the duration of the study. They were told that they would not receive their speech topic until later in the session, and that they would give the speech at the end of the session after a 10‐min preparation period that would occur later on (in the standard TSST, participants would immediately transition to the preparation period after receiving the instructions; Kirschbaum et al., 1993). Next, to ensure that participants perceived sufficient stress prior to the break, a math task was administered. Participants were instructed to serially subtract 13 from 1022 as fast as they could for 5 min, and the examiner provided them with feedback every time they made a mistake. Phase 2 began after a 40‐min break that included a nap or wakefulness. During Phase 2, participants were then given their speech topic for the first time, “what makes you the ideal candidate for the job”, and 10 min to prepare their speech.

### 1.4 Electroencephalography

Electroencephalography (EEG) was recorded from seven electrodes embedded in an elastic cap at standard 10–20 system sites (F3, F4, Fpz, C3, C4, O1, O2) referenced to the average of the mastoids, with Fz as the ground electrode. Additional electrodes were applied beside each eye to detect eye movements and across the chin to measure muscle tension to aid in sleep staging. All electrode impedances were ≤ 10 kΩ. Data were recorded and amplified with a 250‐Hz sampling rate, and bandpass filtered between 0.25 and 0.100 Hz.

### 1.5 Procedure

Upon arrival, participants gave written, informed consent, and then nap participants were prepped for EEG recording. Next, both groups completed a set of demographic questions, the sleep questionnaires (PSQI, KSD), and the SSAI and PANAS to assess “baseline” perceived stress and affect. Acute stress was then triggered by completing Phase 1 of the modified TSST.

Participants then completed the SSAI and PANAS again to assess perceived stress and affect, and working memory was assessed with the Digit Span‐Backwards test (the “after stressor” assessments). All participants then took a 40‐min break. Nap participants slept in a quiet bedroom while EEG was recorded. Wake participants watched a video describing manufacturing processes and answered 17 simple questions about the video. This neutral task ensured that participants stayed awake but did not experience additional stressors during the break.

After the break, perceived stress, affect and working memory were again assessed with the SSAI, PANAS and the Digit Span‐Backwards, respectively (the “after break” assessments). Next, acute stress was reinforced by completing Phase 2 of the modified TSST. Perceived stress and affect were then assessed with the SSAI and PANAS, respectively, for a final time (the “after preparation period” assessments). Participants were then informed that they did not need to give the speech and were debriefed.

### 1.6 Statistical analyses

Sleep staging and computations of spectral power were completed with Prana software (PhiTools). Sleep data were scored using standard scoring criteria (Iber et al., 2007), and EEG spectral power was computed following visual artefact rejection by applying a fast Fourier transform with a Hanning function in 4‐s intervals with 50% overlap, providing a frequency resolution of 0.25 Hz. One participant was excluded from the spectral analysis for excessive artefacts in the EEG recording. Power estimates of SWA (0.5–1.5 Hz) and sigma activity (12–15 Hz) were averaged across 30‐s epochs corresponding to the epochs used for sleep staging. SWA was selected for further analyses based on prior reports of its involvement in cognition and synaptic downscaling (Tononi & Cirelli, 2006; van der Werf et al., 2011). Estimates of SWA were averaged across all sleep periods at F3, F4 and Fpz electrodes as SWA is maximal over frontal recording sites (Massimini et al., 2004). EEG data from F4 were not available for one participant due to a bad recording electrode at this site, and thus data were averaged across F3 and Fpz only for this participant. Sigma activity was also analysed, given its previously reported relationship with mood (Jones et al., 2019). Sigma power was averaged across C3, C4, F3, F4 and Fpz electrodes during stage 2 sleep, as sigma is most robust at these sites and most prevalent during stage 2 (De Gennaro & Ferrara, 2003).

IBM SPSS Statistics version 27 for Windows was used for all other analyses. Potential outliers were identified as scores greater than 3 standard deviations above or below the group mean for each outcome measure (perceived stress, negative affect and working memory) at each time point, and all analyses were run with and without outliers (when present). Analyses of variance (ANOVA) were used to examine the effects of time, group and sex on the outcome measures, and post hoc independent and paired‐samples *t*‐tests were conducted as follow‐up tests when necessary. Two‐tailed Pearson's correlations were also conducted to identify potential relationships between the outcome variables and sleep stages and spectral power, using a Bonferroni correction for multiple comparisons. An alpha level of 0.05 was used for all statistical analyses.

## 3 RESULTS

### 3.1 Sleep measures

Average global PSQI scores revealed slightly poor sleep quality during the past month for both groups (nap group: M = 5.92, SD = 1.89; wake group: M = 6.03, SD = 2.49). An independent sample *t*‐test indicated there was no difference in PSQI scores between groups (*t*_62_ = 0.18, *p* = 0.86). For PSQI scores, one outlier was found in the wake group and one outlier was found in the nap group. However, when the analysis was repeated excluding the outliers, the comparison was still not significant (*t*_60_ = 0.63, *p* = 0.53).

The KSD responses indicated that participants were not sleep deprived, reporting an average of 7.00 hr of sleep the night before the study (SD = 1.69). Independent samples *t*‐tests between nap and wake groups were conducted on responses to the six KSD questions regarding sleep quantity. Group differences were only observed for responses to the sleep latency question (Table 1, question 4), indicating that sleep latency was shorter for the nap (M = 14.00 min, SD = 14.55) than the wake group (M = 26.05 min, SD = 25.33; *t*_65_ = −2.26, *p* = 0.027, *d* = −0.56). However, an outlier was identified in the wake group, and the group difference was no longer significant after excluding the outlier and repeating the analysis. A general sleep quality score was computed by averaging scores across the seven KSD questions regarding sleep quality. A *t*‐test indicated there was no difference in sleep quality between nap and wake groups (*t*_68_ = −0.8, *p* = 0.94).

Sleep latency, time spent in each sleep stage, and wake after sleep onset are reported in Table 2. Nap participants included in analyses slept for a minimum of 11.5 min and a maximum of 35.5 min during the break (M = 25.9 min, SD = 6.63). Mean SWA was 232.84 μV^2^ (SD = 172.80). Previous research suggests that measures of SWA can differ between hemispheres on the first night of sleeping in a new environment (Tamaki et al., 2016). However, no differences between right and left hemisphere SWA (measured at F4 and F3 electrodes, respectively) were present (*t*_26_ = 0.28, *p* = 0.78). Mean sigma activity was 12.40 μV^2^ (SD = 8.44), and also did not differ between hemispheres (*t*_27_ = 0.84, *p* = 0.41).

**TABLE 2:** Average time spent in each sleep stage (min)

TST	Stage 1	Stage 2	SWS	REM	Latency	WASO
25.88 (6.63)	4.47 (2.44)	14.29 (6.19)	7.07 (7.40)	0.0 (0.0)	9.66 (6.73)	4.53 (5.60)

*Note*: Standard deviations in parentheses. The sleep window was 40 min.

Abbreviations: Latency, sleep‐onset latency; REM, rapid eye movement sleep; SWS, slow‐wave sleep; TST, total sleep time; WASO, wake after sleep onset.

### 3.2 Perceived stress

**FIGURE 2:**
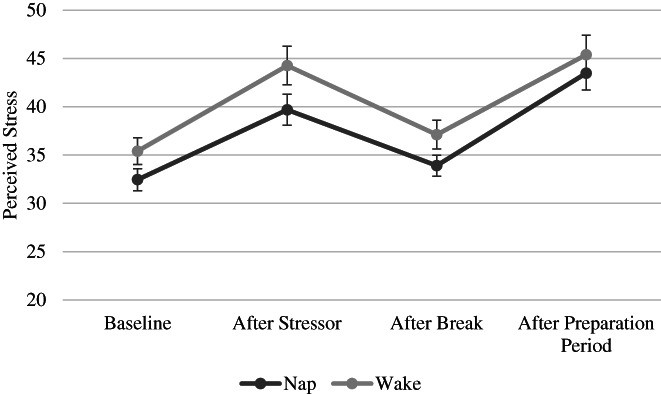
No differences in perceived stress were observed across groups (nap, wake) at the four time points (baseline, after stressor, after break, after preparation period). Bars represent standard error of the mean

Preliminary analyses did not identify any outliers in SSAI scores. A 4 × 2 × 2 ANOVA was conducted on SSAI scores with time (baseline, after stressor, after break, after preparation period), group (nap, wake) and sex (male, female) as independent variables (Figure 2). The break reduced perceived stress levels in both groups (*F*_3,66_ = 41.67, *p* < 0.001). Paired‐sample *t*‐tests indicated lower SSAI scores at baseline (M = 34.19, SD = 7.92) and after the break (M = 35.79, SD = 8.31) compared with after the stressor (M = 42.37, SD = 11.39) and after the preparation period for all participants (M = 44.60, SD = 11.49; all *p‐*values < 0.001).

In addition, females and males differed in perceived stress levels at some, but not all, time points (*F*_3,62_ = 2.87, *p* = 0.043). There were no sex differences in SSAI scores at baseline or after the break (*t*_68_ = −0.35, *p* = 0.73, *t*_68_ = −0.85, *p* = 0.40, respectively). However, females had higher SSAI scores than males after the stressor (*t*_68_ = −2.11, *p* = 0.039, *d* = 0.50) and after the preparation period (*t*_68_ = −2.07, *p* = 0.043, *d* = 0.49; Table 3). Perceived stress after the break did not differ from perceived stress at baseline for both males (*t*_34_ = −1.15, *p* = 0.257) and females (*t*_34_ = −1.35, *p* = 0.19), and both males and females showed reductions in perceived stress after the break compared with after the stressor (males: *t*_34_ = 3.675, *p* < 0.001; females: *t*_34_ = 4.54, *p* < 0.001), although this reduction was larger in females (*t*_68_ = 2.58, *p* = 0.012, *d* = 0.62). No other significant effects or interactions were present (*p*‐values > 0.1).

**TABLE 3:** Mean perceived stress scores by time and sex

	Baseline	After stressor*	After break*	After preparation period
Males	33.86 (8.01)	39.57 (11.26)	34.94 (8.60)	41.83 (11.48)
Females	34.51 (7.94)	45.17 (10.98)	36.63 (8.05)	47.37 (10.98)

*Note*: Standard deviations in parentheses. *Indicates a significant difference between males and females (*p* < 0.05).

### 3.3 Affect

**FIGURE 3:**
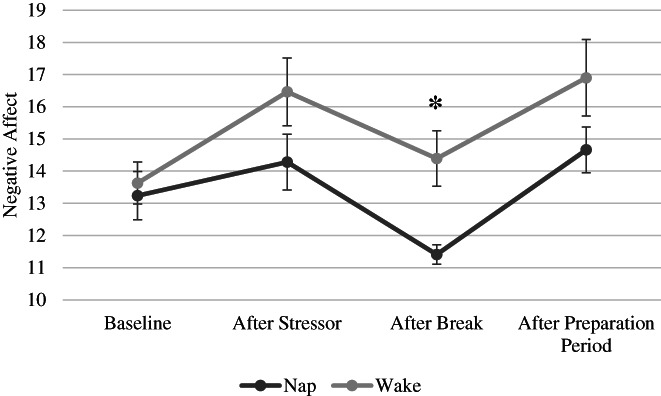
Negative affect scores were lower for participants in the nap group compared with the wake group after the break but not at other time points (baseline, after stressor, after preparation period). Bars represent standard error of the mean (*p < 0.05)

A 2 × 2 × 4 ANOVA was conducted on negative affect scores with time (baseline, after stressor, after break, after preparation period), group (nap, wake) and sex (male, female) as independent variables (Figure 3). Similar to the pattern observed in SSAI scores, negative affect differed across the four timepoints (*F*_3,66_ = 13.31, *p < 0*.001). Paired samples *t‐*tests indicated lower negative affect scores at baseline (M = 13.47, SD = 4.10) and after the break (M = 13.16, SD = 4.57), than after the stressor (M = 15.56, SD = 6.03) and after the preparation period (M = 15.97, SD = 6.38; all *p‐*values < 0.001). In addition, the magnitude of the negative affect reduction from after the stressor compared with after the break differed between the nap and the wake groups (*F*_3,62_ = 3.11, *p* = 0.032). Immediately after the break, the nap group had lower negative affect than the wake group (*t*_68_ = −2.98, *p* = 0.006, *d* = 0.73), whereas no differences in negative affect across groups at baseline, after the stressor or after the preparation period were observed (*p*‐values > 0.05). Although both groups showed lower negative affect after the break than after the stressor (nap group: *t*_28_ = 3.70, *p* < 0.001, *d* = 0.69; wake group: *t*_40_ = 2.73, *p* = 0.009, *d* = 0.43), only the nap group showed reduced negative affect compared with the baseline level of negative affect (*t*_28_ = 2.85, *p* = 0.008, *d* = 0.53; wake group: *t*_40_ = 1.24, *p* = 0.22). No other main effects or interactions were present (all *p‐*values > 0.05).

One outlier was present in the wake group after the stressor, after the break and after the preparation period, and one outlier in the nap group was present after the preparation period. Therefore, the analyses of negative affect described above were conducted again omitting the outliers. The same pattern of results was observed.

### 3.4 Working memory

**FIGURE 4:**
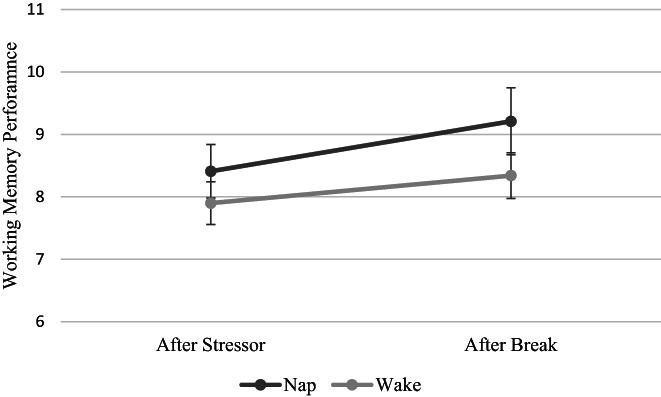
No differences in working memory performance were observed across time (after stressor, after break) or across group (nap, wake). Bars represent standard error of the mean

A 2 × 2 × 2 ANOVA was conducted on Digit Span‐Backwards scores with time (after stressor, after break), group (nap, wake) and sex (male, female) as independent variables (Figure 4). Working memory was higher after the break (M = 8.70, SD = 2.60) than after the stressor (M = 8.11, SD = 2.24; *F*_1,68_ = 9.87, *p* = 0.003). No other effects or interactions were significant (all *p‐*values > 0.1).

One outlier in the wake group was identified in Digit Span‐Backwards scores after the stressor. However, when analyses were repeated excluding the outlier, the same pattern of results was present.

### 3.5 Percent change

To account for the possibility that small differences between groups at baseline could contribute to results observed at later time points, percent change scores relative to baseline were calculated for after the stressor, after the break, and after the preparation period time points for perceived stress and negative affect. Two 2 × 2 × 3 ANOVAs were conducted with group, sex and time (after the stressor, after the break, and after the preparation period) as independent variables; one for perceived stress and one for negative affect. The pattern of results were the same as those described above when raw scores were used as dependent variables.

### 3.6 Relationships between sleep and stress, affect, and working memory

Pearson's correlations (including Bonferroni correction for multiple comparisons) were conducted to determine if total sleep time, time spent in specific sleep stages, SWA or sigma activity predicted changes in perceived stress, negative affect or working memory across the nap period for nap participants. Change scores were computed for perceived stress, negative affect and working memory by subtracting scores obtained after the nap from scores obtained after the stressor. No significant correlations were observed (all corrected *p*‐values > 0.05). Pearson's correlations were also conducted to determine if total sleep time, time spent in specific sleep stages, SWA or sigma activity were related to negative affect and perceived stress after the stressor, prior to the nap. No significant correlations were observed (all corrected *p*‐values > 0.05).

### 3.7 Included versus excluded nap participants

Given the relatively large number of participants in the nap group that were excluded for failing to achieve 5 min of stable sleep (*n* = 27), additional analyses were conducted to determine if this failure could have resulted from differences in any of the factors measured in this experiment between nap participants who successfully slept versus nap participants that did not. Data from one of the excluded participants could not be recovered due to computer error and thus were omitted from these analyses. Independent samples *t*‐tests indicated that there were no differences in negative affect or perceived stress at baseline or after the stressor between included and excluded nap participants (all *p*‐values > 0.05). Likewise, no differences in working memory before the break were present between groups (*t*_53_ = 0.17, *p* = 0.87). In addition, the included and excluded nap participants did not differ in PSQI scores (*t*_53_ = 1.38, *p* = 0.17) or in responses to any of the KSD questions (all *p*‐values > 0.05). Thus, there are no systematic differences in the variables measured in this experiment that could explain why some nap participants were able to fall asleep whereas others were not.

## 4 DISCUSSION

This study evaluated the potential benefit of a brief daytime nap during an acute stressor on perceived stress, negative affect and working memory in a non‐sleep‐deprived sample. Perceived stress decreased to a similar extent in the nap and the wake groups after the break, indicating that a 40‐min nap does not provide an additional benefit for reducing perceived stress beyond taking a break. Similarly, working memory improved in both groups following the break, suggesting that a brief nap during an acute stressor does not confer additional benefits for working memory beyond a break containing wakefulness. However, negative affect was lower after the break in the nap group relative to the wake group, and only the nap group showed a significant reduction in negative affect from baseline. Notably, these effects were fairly robust, as Cohen's *d* measures were greater than 0.5 for these comparisons. In addition, although females had larger increases in subjective stress after an acute stressor than males as has been reported previously (Kelly et al., 2008), sex did not influence the effects of a nap on perceived stress or negative affect. Finally, perceived stress and negative affect increased after the preparation period in both the nap and wake groups, suggesting that the beneficial effect of napping for negative affect does not persist after a stressor is reintroduced.

These results are the first to demonstrate that after the induction of acute psychosocial stress, a 40‐min nap can improve negative affect relative to a comparable time spent awake. In a paradigm that did not include acute stress, Jones et al. (2019) found that longer, 120‐min naps can improve negative affect, and this improvement was predicted by time spent in stage 2 sleep and non‐REM sigma power density. The current results extend this finding by demonstrating that even 40‐min naps can reduce negative affect, and that the deleterious effects a stressor may have on subsequent sleep are not enough to eliminate the benefits for negative affect (Ackermann et al., 2019). Although reduced negative affect was not related to time spent in stage 2 sleep in the current study, it should be noted that Jones et al. (2019) used a slightly different measure of affect (ratio of positive to negative affect), which, along with a longer nap duration, likely influenced the extent of the relationship observed in their study. Regardless, the current results suggest a brief nap may be more beneficial than merely a break to reduce negative affect brought on by an acute stressor.

Although it was hypothesized that a brief nap may reduce perceived stress, it was reduced to a similar extent in both nap and wake groups. One possibility for the failure to find an additional reduction in perceived stress in the nap group compared with the wake group concerns the brief amount of time participants were allowed to sleep (40 min). Previous studies have demonstrated that psychological stress can reduce the amount of time spent in both SWS and REM, reduce sleep efficiency, and increase sleep latency (Ackermann et al., 2019; Germain et al., 2003; Gross & Borkovec, 1982). If SWS is necessary to reduce the stress response by recalibrating synaptic strengths and optimizing cognitive performance (Tononi & Cirelli, 2006; Van der Werf et al., 2011), it is possible that participants did not spend enough time in SWS to procure the benefits that SWS may have for perceived stress. Supporting this possibility, the average time spent in SWS for participants in the nap group was only 7 min, and 10 participants failed to achieve any time in SWS. Another possible explanation for the inability of the nap to reduce perceived stress more so than wakefulness is that due to the brief nature of the nap, no participants obtained REM sleep. Reduced overnight REM has been associated with elevated stress responses the following day, suggesting REM contributes to the stress‐reducing benefits of sleep (Motomura et al., 2017). Thus, it may be the case that reductions in perceived stress only occur following longer naps in which REM is achieved. A final possibility is that neither the neutral video nor the nap was therapeutic, but instead they merely served as temporary distractions from the impending stressful task. The increase in perceived stress after the preparation period was roughly equivalent to the increase when the stressor was initially introduced, suggesting the presence of dichotomous states, where participants were either anxious/stressed or not. The break may have acted as a stress distractor and brought perceived stress back to the baseline “not anxious” state.

The results of this study may also have important implications for researchers interested in modifying the TSST. Specifically, the temporary nature of the reduction in perceived stress in the current study also indicates that the attentional focus of participants during the break may be an important determinant of how a break will influence stress. Young and Nolen‐Hoeksema (2001) found that after an hour‐long preparation period in the TSST, cortisol responses were strikingly diminished. Thus, as in the current experiment, an hour passed between the introduction of the stressor and the stressful event, but constant attention to the stressor in Young and Nolen‐Hoeksema (2001) diminished the stress response. However, it should be clarified that objective measures of stress (e.g. cortisol levels) were not obtained in the current study. Some research indicates that subjective measures accurately track objective measures of stress (Merz & Wolf, 2015; Pearman et al., 2020), although this finding has been inconsistent (Childs et al., 2014; Leininger & Skeel, 2012). Thus, comparisons with other studies using only objective stress measures may be limited. Nonetheless, exploring how attention to a stressor during delay periods in the TSST may affect both subjective and objective stress may be an interesting avenue for future research.

In addition, although no sex difference in perceived stress was observed at baseline or after the break, perceived stress was greater in females than males both after the stressor and after the preparation period. Some evidence suggests that women have elevated subjective negative emotional responses, specifically increased fear and irritability, to the TSST compared with men (Kelly et al., 2008), and that stress may be more likely to evoke anxiety in women than men (Sandanger et al., 2004). While sex differences in negative affect were not observed here, the initial increase in perceived stress after the stressor was introduced was almost twice as large in females than in males, supporting the notion that women may experience greater perceived stress in response to social stressors than men. However, it is interesting to note that after the break, perceived stress for females and males was not different from baseline levels, indicating that the break, including either wakefulness or a nap, reduced stress to a greater extent in females than males.

Working memory increased across the break for both the nap and wake groups. It is unlikely that this improvement is merely a practice effect, as Digit‐Span Backwards has strong test–retest reliability (Catron, 1978). Instead, this improvement may be attributable to the stress decrease afforded by the break, as stress can impair working memory (Schoofs et al., 2008). If this hypothesis is correct, working memory would be expected to be higher at baseline than after the stressor, and likewise it should decrease alongside the exacerbation of perceived stress seen after the preparation period. Unfortunately, these predictions cannot be verified as the present study design did not include a measure of working memory at baseline or after the preparation period due to time constraints. Alternatively, working memory improvements after a nap may require REM sleep, as suggested by Lau et al. (2015), or longer amounts of non‐REM sleep than afforded by a 40‐min nap. Regardless, it appears that a brief nap during an acute stressor is not sufficient to improve working memory beyond a comparable break filled with wakefulness.

In addition to only obtaining measures of working memory immediately before and after the break, other limitations of this study should be noted. First, participants were still undergoing stress when the break occurred, as they were anticipating giving a speech after the break. The choice to schedule the nap in the middle of the stressful period was made to examine how sleep may influence an individual's ability to cope with perceived stress rather than to examine how sleep contributes to stress recovery. However, it is possible that ongoing anticipatory stress could have influenced the measures obtained after the break. For example, the ongoing stress may have reduced the magnitude of the increase in working memory performance or the improvement in negative affect and/or perceived stress observed after the break. Therefore, comparisons of the current results with other research in which the stressor is not ongoing may be limited.

Another limitation is that participants in the nap group did not have an adaptation nap prior to the experiment to acclimate to sleeping in a novel environment while wearing electrodes. This decision was purely practical due to resource constraints. However, this may have contributed to the high number of participants in the nap group (27) who were excluded for not being able to achieve 5 min of stable sleep. Tamaki et al. (2016) observed that participants sleeping in a novel environment exhibited hemisphere asymmetries in SWA, which could be indicative of increased vigilance when sleeping in a new place. In the current study, no hemisphere differences in SWA activity were observed, suggesting that the participants who fell asleep may have been less susceptible to increases in vigilance in the novel environment. Alternatively, it is possible that when sleeping in a novel environment, hemisphere asymmetries present during overnight sleep (as in Tamaki et al., 2016) are not apparent during an afternoon nap. Additional research would be necessary to test this possibility. Another factor that may have made it more difficult for participants to fall asleep in the laboratory was the fact that the stressor had already been introduced prior to the nap, and psychosocial stress can increase sleep latency (Ackermann et al., 2019). In this way, the nap participants who fell asleep may not be representative of the population, and instead may reflect individuals who are less susceptible to stress‐related sleep disturbances.

One big difference between the nap and wake groups is that the nap group slept with electrodes on whereas the wake group did not wear any electrodes during the break. It is possible that this difference could have influenced the observed results. However, arguing against this possibility, electrodes were applied to participants in the nap group before the experiment began, before baseline measures of perceived stress and negative affect were obtained, and no differences in these baseline measures were observed.

A final factor that may have influenced the working memory results in particular is that participants in the wake group were asked to answer questions about the video they watched during the break as the video was playing. This may have placed cognitive demands on these participants that could have diminished working memory performance after the break. The questions were very simple and minimized cognitive effort, but the possibility that answering these questions impacted working memory performance cannot be ruled out.

A 40‐min nap before approaching a stressful task does not dampen perceived stress or improve working memory to a greater extent than taking a 40‐min break. This could be due to the absence of REM sleep during brief naps or insufficient SWS in such a short time period. Exploring the effects of longer naps (e.g. 90 min) on stress and working memory may help to disentangle the role of different sleep stages in reducing stress responses and improving cognition. Nonetheless, napping after an acute stressor does appear to be an effective way to reduce negative affect, alleviating the emotional distress that commonly occurs during acute stress. Reducing negative affect during stressful experiences could reduce the risk of burnout and increase physiological and psychological health. Thus, taking a brief nap represents a simple and time‐effective approach to controlling negative emotional responses to acute stress which, if employed consistently, might ultimately contribute to the reduction of chronic effects of negative emotions on our bodies and minds.

## AUTHOR CONTRIBUTIONS

N. Wofford and C.E. Westerberg developed the study concept and design. Data collection and analyses were completed by N. Wofford, and C.E. Westerberg and N. Ceballos contributed to data interpretation. All authors were involved with drafting the manuscript and provided revisions. All authors approved the final version of the manuscript for submission.

## CONFLICT OF INTEREST

The authors declare that there is no conflict of interest.

## Data Availability

The data that supports the findings of this study are openly available in figshare at https://doi.org/10.6084/m9.figshare.13012817.v1.
